# TNF-α is involved in activating DNA fragmentation in skeletal muscle

**DOI:** 10.1038/sj.bjc.6600167

**Published:** 2002-03-18

**Authors:** N Carbó, S Busquets, M van Royen, B Alvarez, F J López-Soriano, J M Argilés

**Affiliations:** Cancer Research Group, Departament de Bioquímica i Biologia Molecular, Facultat de Biologia, Universitat de Barcelona, Diagonal 645, 08028-Barcelona, Spain

**Keywords:** DNA fragmentation, skeletal muscle, TNF-α, cancer cachexia, apoptosis

## Abstract

Intraperitoneal administration of 100 μg kg^−1^ (body weight) of tumour necrosis factor-α to rats for 8 consecutive days resulted in a significant decrease in protein content, which was concomitant with a reduction in DNA content. Interestingly, the protein/DNA ratio was unchanged in the skeletal muscle of the tumour necrosis factor-α-treated animals as compared with the non-treated controls. Analysis of muscle DNA fragmentation clearly showed enhanced laddering in the skeletal muscle of tumour necrosis factor-α-treated animals, suggesting an apoptotic phenomenon. In a different set of experiments, mice bearing a cachexia-inducing tumour (the Lewis lung carcinoma) showed an increase in muscle DNA fragmentation (9.8-fold) as compared with their non-tumour-bearing control counterparts as previously described. When gene-deficient mice for tumour necrosis factor-α receptor protein I were inoculated with Lewis lung carcinoma, they were also affected by DNA fragmentation; however the increase was only 2.1-fold. These results suggest that tumour necrosis factor-α partly mediates DNA fragmentation during experimental cancer-associated cachexia.

*British Journal of Cancer* (2002) **86**, 1012–1016. DOI: 10.1038/sj/bjc/6600167 www.bjcancer.com

© 2002 Cancer Research UK

## 

Tumour necrosis factor-α (TNF) is a cytokine synthetised and released by blood monocytes and tissue macrophages in response to invasive stimuli, which exerts diverse metabolic effects (see Evans *et al*, 1989; Argilés *et al*, 1997 for a review). Although a large body of evidence suggests that this cytokine participates in the protein wasting and loss of nitrogen associated with cachectic situations (Argilés *et al*, 1997; Argilés and López-Soriano, 1999), the mechanisms underlying such actions still remain obscure. Both in man and mouse, TNF binds as homotrimer to two kinds of receptors, TNFR1 (p55) and TNFR2 (p75) (Hohman *et al*, 1989); in addition, lymphotoxin-α (LTα or TNF-β) also binds to the same receptors. The pleiotropic functions of TNF can be partially explained by the presence of its receptors in almost all types of nucleated cells. The expression of the genes encoding the two receptors is differentially regulated in different cells. TNFR1 is expressed constitutively and plays a central role in many biological processes, whereas less is known about TNFR2 expression (see Vandenabeele *et al*, 1995 for a review).

Cancer cachexia is perhaps the most common manifestation of advanced malignant disease. Cachexia occurs in the majority of cancer patients before death, and is responsible for the death of 22% of these patients (Warren, 1932), although lower percentages have been considered in more recent publications (Dworzak *et al*, 1998). The abnormalities associated with cancer cachexia include anorexia, weight loss, muscle loss and atrophy, anaemia and alterations in metabolism (see Argilés *et al*, 1997, for review). The degree of cachexia is inversely correlated with the survival time of the patient and it always implies a poor prognosis (Harvey *et al*, 1979; Nixon *et al*, 1980; De Wys, 1985). Perhaps asthenia is one of the most relevant characteristics of cachexia, reflecting the extensive muscle waste that takes place in the cachectic cancer patient (Argilés *et al*, 1992), and is also characterised by a general weakness as well as physical and mental fatigue (Adams and Victor, 1981). Actually, body protein depletion is one of the main trends of cachexia and it involves not only skeletal muscle but it also affects cardiac proteins, resulting in alterations in heart performance (Drott *et al*, 1986).

Apoptosis, the programmed type of cell death, is an important physiological process in the development and homeostasis of multicellular organisms. Apoptotic cell death is characterised by a common pattern of morphological alterations such as chromatin condensation, membrane blebbing, DNA fragmentation and cell shrinkage (Huppertz *et al*, 1999). In cardiac muscle, apoptosis has been recognised as a component of many common pathologies including chronic heart failure, cardiac sudden death, viral myocarditis and ischaemia (Tanaka *et al*, 1994; Itoh *et al*, 1996; Kajstura *et al*, 1996). Moreover, during chronic heart failure, rat skeletal muscle atrophy has been related to apoptosis (Dalla Libera *et al*, 1999). Indeed, apoptosis has already been described associated with skeletal muscle atrophy (Allen *et al*, 1997; Tews and Goebel, 1997; Tews *et al*, 1997) and other diseases (see Sandri and Carraro, 1999, for review).

During cancer cachexia, the activation of the ubiquitin-dependent proteolytic pathway seems to be responsible for the muscle protein mobilisation (Argilés and López-Soriano, 1996). Recently, a link between the apoptosome and the proteasome pathway has been described (Dimmeler *et al*, 1999). In addition, we have demonstrated that during experimental cancer cachexia, DNA fragmentation is increased in skeletal muscle (Van Royen *et al*, 2000). Therefore, it was the aim of the present investigation to examine if cytokines, TNF in particular, synthetised during the evolution of the tumour-induced cachectic process, were involved in the changes in skeletal muscle DNA content and integrity.

## MATERIALS AND METHODS

### Animals, tumour inoculation and TNF treatment

Male C57BL/6 mice (Criffa, Barcelona, Spain) weighing about 25 g were used. Mice were divided into two groups, namely controls and tumour hosts. The latter received an intramuscular (left thigh) inoculum of 5×10^5^ Lewis lung carcinoma cells obtained from exponential tumours. The development of a nodule at the side of the injection, growing in size up to almost 20% of the animal weight in 2 weeks, was considered as an index of effectiveness of the inoculation. Nearly 100% of the injected animals developed hind-leg tumours, and all of them were affected by lung metastasis from day 7 onwards. On day 15 after tumour transplantation, animals were weighed and anaesthetised with ketamine/xylacine (Imalgene and Rompun respectively).

Concerning TNFRI-deficient mice, homozygous mice for a disrupted *Tnfr*I allele (*Tnfr*I°) were used. The gene targeting vector consisted of a genomic mouse DNA fragment (Rothe *et al*, 1993), in which exons 2 and 3 and part of exon 4 of the *Tnfr*I gene were replaced by a *neo* cassette. This deletion disrupts the gene and removes the coding information for the cysteine-rich domains I and II of the receptor, which have been shown to be essential for ligand binding (Banner *et al*, 1993). Germ-line transmitters of the mutated *Tnfr*I allele were crossed with C57BL/6 mice and the resulting heterozygous mice interbred to yield homozygous mutant offspring. The F1 generation displayed the expected mendelian 1 : 2 : 1 of wild-type (+/+), heterozygous (0/+) and homozygous (0/0) mutant mice, indicating that *Tnfr*I expression is not required for normal embryonic development.

In the experiments involving chronic TNF treatment, female Wistar rats (*Interfauna Iberica*) weighing 100–150 g were used. TNF was given intraperitoneally for 8 days at a dose of 100 μg kg^−1^ per day (two administrations at 08:00 and 20:00 h). Control animals received 0.5 ml of vehicle (physiological saline). Human recombinant-derived TNF-α (specific activity 8.1×10^6^ u mg^−1^ protein, purity >99% containing less than 0.137 ng mg^−1^ endotoxin) was generously given by BASF/Knoll AG (Ludwigshafen, Germany).

All animals were maintained on a regular light-dark cycle (light on from 08:00 to 20:00 h) and had free access to food and water. The diet (BK Universal GJ/SL, Sant Vicenç del Horts, Barcelona, Spain) consisted of 45.5–48.5% carbohydrate (3.5% absorbible glucose, 43–45% starch), 18.5% protein and 3.1% fat (the residue was non-digestible material).

All animal experiments have been carried out with ethical committee approval. The ethical guidelines that were followed meet the standards required by the UKCCCR guidelines (Workman *et al*, 1998).

### Biochemicals

They were all reagent grade and obtained either from Roche (Barcelona, Spain) or from Sigma Chemical Co. (St Louis, MO, USA).

### DNA and protein content

Samples of skeletal muscles were homogenised in an ammonium hydroxide/Triton X-100 extraction buffer (supplemented with protease inhibitors) and used for the determination of both protein (Bradford, 1976) and DNA (Downs and Wilfinger, 1983) content.

### DNA fragmentation assay

Gastrocnemius muscles were homogenised and incubated at 48°C overnight in Kauffman buffer (0.5 M TRIS, 2 mM EDTA, 10 mM NaCl, 1% SDS) in the presence of 200 μg ml^−1^ of Proteinase K, and DNA was extracted with phenol/chloroform. After ethanol precipitation, the pellets were resuspended and the DNA integrity was checked in a 2% agarose gel electrophoresis and ethidium bromide staining. The percentage of DNA fragmentation was quantified by scanning densitometry. Liver from 8-h anti-Fas antibody-treated mice (Ogasawara *et al*, 1993) was used as a positive control of DNA fragmentation.

### Plasma TNF levels

Circulating TNF was evaluated by a murine immunoassay (Genzyme, Cambridge, MA, USA).

### Statistical analysis

Statistical analysis of the data was performed by means of the Student's *t*-test.

## RESULTS

As can be seen in Table 1

**Table 1:**
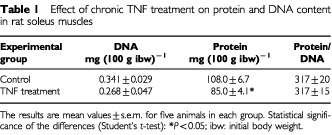
Effect of chronic TNF treatment on protein and DNA content in rat soleus muscles

, chronic administration of TNF to rats for 8 consecutive days results in a decrease (21%) in skeletal muscle protein content (soleus). The cytokine also induces a similar decrease in muscle DNA content although the results do not reach statistical significance. Interestingly, the protein/DNA ratio is unchanged as a result of TNF treatment. At this point, it seemed that DNA was probably being degraded at a faster rate as a consequence of TNF treatment. As can be seen in Figure 1

**Figure 1:**
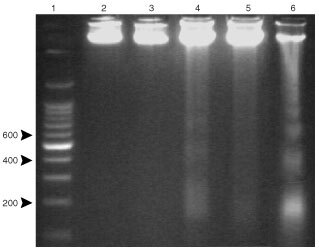
DNA laddering in rats chronically treated with hrTNF-α (100 μg kg^−1^ body weight/day). Lane 1: DNA molecular weight marker; lanes 2–5: 40 μg of gastrocnemius muscle DNA from control (lanes 2 and 3) and TNF-treated (lanes 4 and 5) rats respectively; lane 6: 20 μg of liver DNA from anti-Fas-treated mice (positive control) The percentage of DNA fragmentation was quantified by scanning densitometry.

, DNA fragmentation is clearly induced by the cytokine; indeed it caused an increase of 4.6-fold over the basal fragmentation observed in the control animals. These data agree with our previous report (Van Royen *et al*, 2000) demonstrating that during experimental cancer cachexia, DNA fragmentation was an important event in skeletal muscle. For this reason, we decided to investigate if TNF was involved in this apoptotic event, since tumour-bearing animals generally show high circulating levels of the cytokine (Costelli *et al*, 1993). Bearing all this in mind, in the following experiments we used gene-deficient mice for TNFRI protein (TNFRI KO). As can be seen in Figure 2

**Figure 2:**
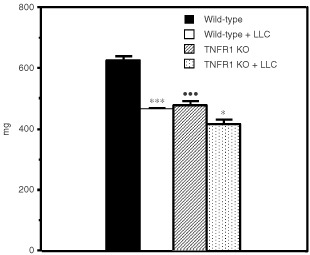
Effects of tumour growth on gastrocnemius weight in tumour-bearing mice. Results are mg (100 g ibw^−1^) of initial body weight. Statistical significance of the results: *P<0,05; ***P<0.001 (*vs* non-tumour); ^•••^*P*<0.001 (*vs* wild-type) *n*=5 for wild-type and KO, *n*=6 for tumour-bearing mice. LLC: Lewis lung carcinoma.

, tumour growth induced an important decrease in gastrocnemius weight (26%) in wild-type animals. In the gene-deficient mice, however, tumour burden only caused a 13% decrease in gastrocnemius weight (Figure 2). Interestingly, control non-tumour-bearing gene-deficient mice have a significantly smaller muscle mass (21%) as compared with the control wild-type mice (Figure 2). Similarly, gastrocnemius total protein content was clearly decreased (29%) by tumour burden in wild-type mice as well as in the TNFRI KO mice (18%) (Table 2

**Table 2:**
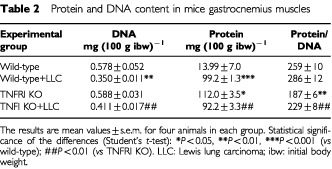
Protein and DNA content in mice gastrocnemius muscles

). The DNA content was also decreased as a result of tumour growth in the wild-type (39%) and in the TNFRI KO mice (30%) (Table 2). Interestingly, the protein/DNA ratio was unchanged in the wild-type mice as a result of tumour burden whereas in the gene-deficient mice tumour growth resulted in a significant increase in this ratio (Table 2).

Figure 3

**Figure 3:**
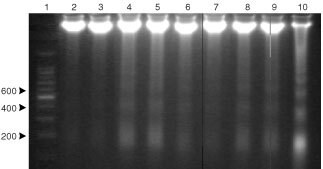
DNA laddering in mice bearing the Lewis lung carcinoma. Lane 1: DNA molecular weight marker; lanes 2–5: 35 μg of gastrocnemius muscle DNA from wild-type control (lanes 2 and 3) and tumour-bearing (lanes 4 and 5) respectively; lanes 6–9: 35 μg of gastrocnemius muscle DNA from TNFRI KO mice, control (lanes 6 and 7) and tumour-bearing (lanes 8 and 9) respectively; lane 10: 20 μg of liver DNA from anti-Fas-treated mice (positive control) The percentage of DNA fragmentation was quantified by scanning densitometry.

shows the result of the DNA fragmentation analysis. The Lewis lung carcinoma induced a marked increase in DNA fragmentation in wild-type mice (9.8-fold). The tumour also induced an increase in DNA fragmentation in the gene-deficient mice but this was much more modest (2.1-fold). Interestingly, control gene knockout mice have a higher rate of DNA fragmentation (3.9-fold) than that observed in the control wild-type mice (Figure 3). The circulating concentrations of TNF are shown in Table 3

**Table 3:**
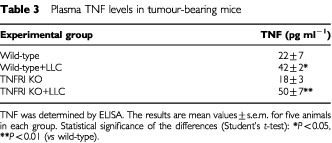
Plasma TNF levels in tumour-bearing mice

. It can be seen that tumour growth results in an increase in circulating cytokine both in wild-type (91%) and gene-deficient (178%) animals.

## DISCUSSION

DNA fragmentation is a common feature of apoptotic cell death and we have previously suggested that the muscle wasting that accompanies cancer cachexia could be linked to an apoptotic phenomenon by which muscle cells lose not only protein but also DNA (Van Royen *et al*, 2000). Apoptosis has already been described in human (Tews and Goebel, 1997; Tews *et al*, 1997) and rat (Dalla Libera *et al*, 1999) atrophic muscle as well as in insect muscle (Schwartz *et al*, 1993). In patients with malignant tumours, anorexia, weight loss, emaciation and progressive alterations of vital functions are common features associated with cancer cachexia (De Wys, 1985). Although in some cases anorexia, gastrointestinal obstruction or malabsorption are responsible for the weight loss of cachectic patients (Balducci and Hardy, 1985), it cannot be wholely attributed to these causes and therefore it has been postulated to be due to a decrease in the energetic efficiency of the cancer patient. Among the factors involved in decreasing the energetic efficiency, skeletal muscle protein turnover seems to have a very significant role as we have previously shown (see Argilés *et al*, 1997, for review). In addition, apoptosis also seems to be present in cachectic muscle in different experimental tumour models (Van Royen *et al*, 2000). The basic aim of the present investigation was to see if the changes that occur in DNA in skeletal muscle during experimental cancer cachexia are linked to TNF. To test this hypothesis we have used two different experimental approximations: chronic TNF administration to healthy rats and experimental cancer cachexia (induced by the Lewis lung carcinoma in mice) in gene-deficient mice for TNFRI.

Different mediators have been suggested to account for cancer-induced cachexia, but basically the presence of both tumoural and humoural (mainly cytokines, TNF in particular) compounds is associated with depletion of fat stores as well as of muscular tissues (Argilés and López-Soriano, 1999). In fact, the balance between pro-inflammatory cytokines, their soluble receptors and the anti-inflammatory cytokines plays a key role in the development of the cachectic syndrome (Argilés and López-Soriano, 1998). Our research group has demonstrated that TNF is involved in the activation of the ubiquitin-dependent proteolysis that takes place during tumour growth (García-Martínez *et al*, 1994; Llovera *et al*, 1996, 1998). We clearly show here that TNF is also involved in triggering DNA fragmentation in muscle during cancer cachexia, mainly through the TNFRI. Indeed, chronic administration of recombinant human TNF, which can only bind rat TNFRI, clearly induces DNA fragmentation, and the use of a tumour model (where the levels of circulating TNF are highly increased) confirm this fact.

Indeed, the Lewis lung carcinoma is a cachectic tumour that induces an important decrease in body weight without significant changes in food intake, at least in the two first weeks of tumour growth (Llovera *et al*, 1998). Because TNFRI is absent from the cells of these animals, the data obtained here suggest that TNF can be involved in the muscle apoptotic mechanisms triggered by tumour growth through its binding with the TNFRI. However, TNFRI is not the sole receptor responsible for transduction of the death signal, even though it is the most important one. Under certain circumstances, TNFRII also either enhances the TNFRI death signal or, indeed, mediates death independently (Declercq *et al*, 1998; Haridas *et al*, 1998; Weiss *et al*, 1998). The mechanism of the TNFRII death signal has not been characterised. The results clearly show that in the gene-deficient mice apoptosis is not induced by tumour growth to the same extent as in the wild-type animals. In fact, TNF has been shown to trigger apoptosis in many cell types (Obeid *et al*, 1993; Ohta *et al*, 1994; Sidoti-de-Fraisse *et al*, 1998) including cardiac muscle (Krown *et al*, 1996). In addition, a possible link between TNF and apoptosis has already been reported in ‘fast’ skeletal muscles in chronic heart failure (Dalla Libera *et al*, 1999). Interestingly high circulating levels of TNF are detectable in both rat (Costelli *et al*, 1993) and mouse (Llovera *et al*, 1998) tumour models used in this study.

Furthermore, TNF-treatment induces Bcl-2 dephosphorylation, targeting this anti-apoptotic protein for degradation by the ubiquitin proteolytic system (Dimmeler *et al*, 1999). Thus, TNF-induced apoptosis could be mediated by different cellular responses (Liu *et al*, 1996), which include the activation of TNFRI death domain and, as a consequence, the caspase cascade and amplification of this apoptotic pathway by means of ubiquitin-dependent Bcl-2 degradation. Nevertheless, TNF binding to its receptors also induces cell proliferation and survival signals mediated by Bcl-2 activation of NF-κB (Liu *et al*, 1996; Wang *et al*, 1996). Therefore, cell survival depends on a delicate balance between the different TNF signalling pathways. For this reason, future investigations in our laboratory will concentrate on ascertaining the role of this and other cytokines in the activation of the apoptotic process associated with cancer cachexia in skeletal muscle.
